# Unveiling the Anti-Cholera and Active Diabetic Renoprotective Compounds of Maqian Essential Oil: A Computational and Molecular Dynamics Study

**DOI:** 10.3390/molecules28247954

**Published:** 2023-12-05

**Authors:** Mahmoud Dahab, Ping Zhang, Samiah Hamad Al-Mijalli, Emad M. Abdallah

**Affiliations:** 1 Department of Microbiology, Faculty of Pure and Applied Sciences, International University of Africa, P.O. Box 2469, Khartoum 12223, Sudan; 2 Center for Integrative Conservation, Yunnan Key Laboratory for the Conservation of Tropical Rainforests and Asian Elephants, Xishuangbanna Tropical Botanical Garden, Chinese Academy of Sciences, Mengla 666303, China; zhangping@xtbg.org.cn; 3 Department of Biology, College of Sciences, Princess Nourah bint Abdulrahman University, P.O. Box 84428, Riyadh 11671, Saudi Arabia; 4 Department of Science Laboratories, College of Science and Arts, Qassim University, P.O. Box 53, Ar Rass 51921, Saudi Arabia; 140208@qu.edu.sa

**Keywords:** essential oil, antibacterial activity, diabetic nephropathy, inflammation, in silico predictions

## Abstract

Cholera is an exceptionally aggressive infectious disease characterized by the potential to induce acute, copious, watery diarrhea of considerable severity and renal inflammation. Diabetic nephropathy is a serious complication of diabetes mellitus that can lead to kidney failure through inflammation; thus, anti-inflammatory agents are promising therapies for diabetic nephropathy. Previous studies have shown that the essential oil of *Zanthoxylum myriacanthum* var. *pubescens* Huang, Maqian essential oil (MQEO), exhibits potent antibacterial, anti-inflammatory, and renoprotective activities in diabetic mice and has emerged as a potential therapeutic drug for the treatment of diabetic nephropathy complications. Therefore, the present study was carried out to screen the potential inhibition of cholera toxin and the diabetic renoprotective activity of MQEO through computational approaches. Twelve chemical constituents derived from MQEO were docked with cholera toxin and the target proteins involved in diabetic nephropathy, namely, TXNIP, Nrf2, and DPP IV, and, subsequently, the predictions of molecular dynamic simulations, the drug-likeness properties, and the ADMET properties were performed. α-terpineol showed high binding affinities toward the cholera toxin protein. For TXNIP, among all the chemical constituents, α-phellandrene and p-cymene showed strong binding affinities with the TXNIP protein and displayed relatively stable flexibility at the hinge regions of the protein, favorable physicochemical properties in the absence of hepatotoxicity, and low cytotoxicity. For Nrf2, α-terpineol exhibited the highest binding affinity and formed a very stable complex with Nrf2, which displayed high pharmacokinetic properties. All compounds had low free-binding energies when docked with the DPP IV protein, which suggests potent biological activity. In conclusion, based on a computational approach, our findings reveal that MQEO constituents have inhibitory activity against cholera toxin and are promising therapeutic agents for suppressing diabetic inflammation and for the treatment of diabetic nephropathy complications.

## 1. Introduction

In the 21st century, there has been growing scientific interest in exploring plants as potential sources of medicinal compounds, particularly in light of the increasing adverse effects associated with synthetic pharmaceuticals [1,2]. In recent years, the prevalence of drug-resistant microorganisms has significantly escalated, posing a substantial threat to the effectiveness of current medications, notably with a focus on Gram-negative bacteria. Therefore, it is of paramount importance to explore alternative approaches to mitigate this global issue [3,4]. *Vibrio cholerae* (*V. cholerae*), a challenging and ancient Gram-negative pathogen, presents a formidable challenge, as there has been a notable surge in the resistance to the antibiotics traditionally prescribed for its treatment worldwide [5]. The pathogenesis of Asiatic cholera, marked by profuse secretory diarrhea, is linked to the interactions between cholera toxin (CT) and the host cell membrane. Specifically, the binding of the B subunit of cholera toxin (CT-B) to ganglioside GM1 on the cell surface is established as a crucial initial step facilitating the translocation of CT across the membrane. Nevertheless, the precise mechanistic details of this process warrant further investigation [6]. The failure to promptly commence appropriate treatment may result in complications, such as acute kidney failure, metabolic acidosis, circulatory failure, arrhythmias, and, ultimately, fatality [7]. It has been documented that certain plants, including *Thymus vulgaris* (Thyme), *Zingiber officinale* (Ginger), *Allium sativum* (Garlic), *Syzygium aromaticum* (Clove), *Zataria multiflora* (Shirazi thyme), *Mentha spicata* (Spearmint), *Satureja bachtiarica* Bunge (Bakhtiari Savory), *Cuminum cyminum* (Cumin), *Camellia sinensis* (Green tea), *Eucalyptus globulus* (Blue gum), *Rosmarinus officinalis* (Rosemary), and *Punica granatum* (Pomegranate), were found to exhibit effectiveness against *V. cholera* infection [8].

Interestingly, diabetic patients who visited clinical facilities following a cholera infection demonstrated poorly managed diabetes mellitus, even during the period of 1–2 months following their recuperation, with a significant proportion of these individuals requiring insulin therapy [9]. Inflammation in the human body is a natural way for it to respond to a variety of internal and external stressors. Inflammation and oxidative stress are considered to be pivotal initiating and promoting factors for diabetic nephropathy (DN) development [10,11]. DN is a common and serious complication of diabetes mellitus (DM) and is considered a serious chronic kidney disease (CKD), which is a leading cause of end-stage renal disease (ESRD). Currently, the incidence of DN has increased worldwide [12]. Therefore, in recent years, an increasing number of molecular mechanisms underlying the pathogenic significance of inflammation in DN have been elucidated [13]. Some medicinal plants have been reported as efficient treatments against diabetic nephropathy (DN), such as *Cinnamon cassia* (Cinnamon), *Curcuma longa* (Turmeric), *Camellia sinensis* (Green tea), *Psidium guajava* (Guava), *Zingiber officinale* (Ginger), *Trigonella foenum-graecum* (Fenugreek), *Phyllanthus emblica* (Gooseberry)*, Piper nigrum* (Pepper), *Coriandrum sativum* (Coriander)*, Ocimum sanctum* (Tulasi), and *Pterocarpus santalinus* (Red sandalwood) [14]. In-depth investigations have been conducted on diverse medicinal plants with a focus on ensuring methodological reliability, isolating bioactive molecules, and performing chemical characterization. The objective of these studies is to advance the development of novel pharmaceutical agents for use as anti-cholera treatments or in the management of diabetic nephropathy (DN) [15,16,17]. In addition, using plants and spices to treat DM and its complications is thought to be safer, more affordable, and natural. Many *Zanthoxylum* species and their phytochemical constituents are used as medicines and spices around the world and have been reported as potential sources of anti-inflammatory [18], antioxidant [19], anti-cancer [20], antibacterial [21], anti-malarial [22], and anti-sickling [23] drugs.

Maqian (or makwhaen), scientifically known as *Zanthoxylum myriacanthum* var. *pubescens* Huang, is a plant of significant botanical and ethnopharmacological importance. It is indigenous to the southern region of Yunnan province in China and has garnered attention for its applications in both traditional medicine and culinary contexts. The traditional usage of this plant encompasses the treatment of conditions such as enteritis, pediatric hepatitis, and colitis [24]. In the realm of scientific investigation, studies have substantiated its efficacy in detoxification, the amelioration of swelling, and the provision of pain relief effects [25,26]. The predominant volatile constituents in the essential oils extracted from Maqian fruit encompass sabinene, limonene, α-pinene, β-myrcene, linalool, and phelladrene as the major phytochemical components [27]. It has also been reported that MQEO is rich of several terpenes (including α-pinene, α-phellandrene, d-limonene, and α-terpineol), which exhibit potent anti-inflammatory properties and have potential for the development of new anti-inflammatory drugs [19,24,28].

Thioredoxin-interacting protein (TXNIP) is a redox-regulating factor that is expressed in various organs and tissues. TXNIP interacts with the antioxidant thioredoxin (TRX) in the physiological state. TXNIP is involved in a number of pathways that can lead to chronic kidney disease progression, including renal oxidative stress, inflammation, and cell death [29]. The activation of TXNIP leads to the dysregulation of autophagy and lipid metabolism in renal tubules and mitochondria, which can contribute to DN development [30]. By contrast, the downregulation of TXNIP and/or interference with its function has been implicated in the progress of DN and is proven to be an effective therapeutic strategy in DN control [31]. Moreover, TXNIP deficiency protects against the progression of DN [32]. Previous studies have demonstrated that the downregulation of TXNIP via MQEO reversed inflammation and oxidative markers in the kidney in a rodent model of type 1 diabetes mellitus [33]. The molecular mechanism behind how MQEO reverses DN through the suppression of TXNIP is not well understood; therefore, the purpose of this study was to assess the interaction between Maqian chemical compositions and TXNIP using a molecular docking approach.

The nuclear factor erythroid 2-related factor 2 (Nrf2) is a transcription factor that is expressed in different tissues, and it has recently emerged as a key player in the body’s ability to upregulate and promote endogenous antioxidant defense mechanisms [34,35]. It has been demonstrated that Nrf2 alleviates inflammation, which plays a role in the development of DN [36]. Furthermore, Nrf2 suppresses the expression of TXNIP. Under a variety of biological stress conditions, Nrf2 activation orchestrates a cascade of defensive responses that aid in the adaptation and survival of redox homeostasis [37]. The mechanisms by which Nrf2 activators work are not fully understood. Therefore, targeting renoprotective activators and oxidative stress suppressors through Nrf2 expression might be a potential and important strategy for treating DN progression.

The dipeptidyl peptidase IV (DPP IV) enzyme exists in various organs, including the kidney, participates in several biological processes, and is responsible for inactivating incretins that stimulate insulin secretion [38,39]. DPP IV and its inhibitors were involved in inflammatory pathways in the DN condition of a type 1 diabetic animal model [40,41]. Thus, phytochemical constituents from medicinal plants could become feasible and effective therapeutic agents for the suppression of renal inflammation through DPP IV inhibition.

The current study aimed to identify the structure and function relationship of phytochemical compounds derived from MQEO by using molecular docking and molecular dynamic simulations of these compounds with four target proteins: TXNIP, Nrf2, DDP IV, and cholera toxin, which were retrieved along with co-crystallized ligands. The outcomes of our computational study serve as a benchmark for identifying and evaluating particular plant-based inhibitors and open up new possibilities for the creation of cutting-edge and more potent anti-cholera and anti-inflammatory drugs.

## 2. Results

### 2.1. Molecular Docking Analysis of Ligands with Target Proteins

**Figure 1:**
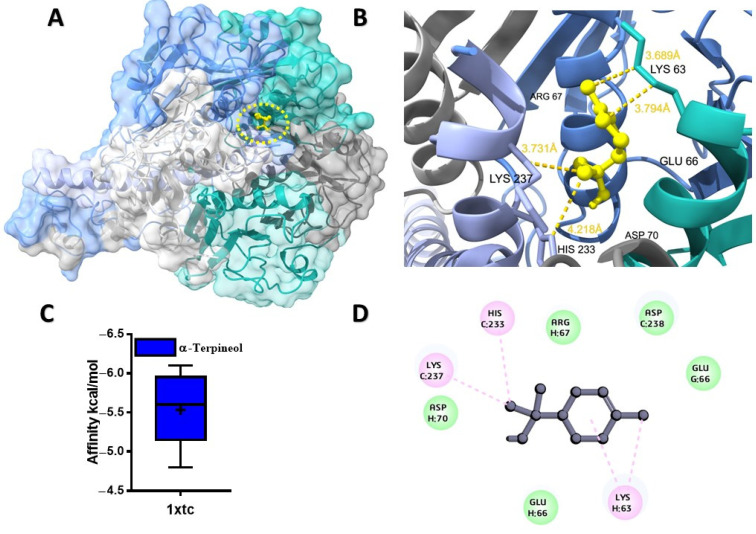
Predicted structures for protein–ligand docked complexes: (**A**) pose view of the interaction of α-terpineol with the cholera toxin protein; (**B**) 3D interaction of α-terpineol with the cholera toxin protein; (**C**) box plot depicting binding affinity scores for predictions of cholera toxin (1xtc) protein with α-terpineol; (**D**) 2D interaction of α-terpineol with key residues.

**Figure 2:**
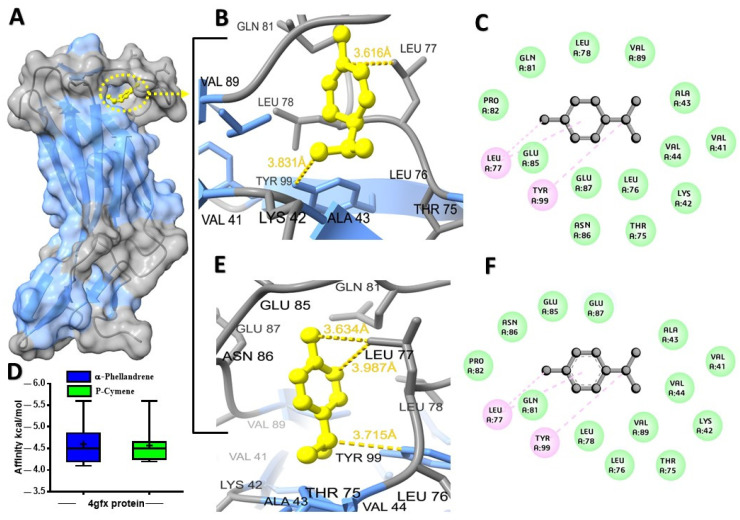
Predicted structures for protein–ligand docked complexes: (**A**) pose view of the interaction of α-phellandrene and p-cymene with TXNIP protein; (**B**) 3D interaction of α-phellandrene with TXNIP protein; (**C**) 2D interaction of α-phellandrene with key residues; (**D**) box plot depicting binding affinity scores for predictions of α-phellandrene (blue) and p-cymene (green) with TXNIP (4gfx) protein; (**E**) 3D interaction of p-cymene with TXNIP protein; (**F**) 2D interaction of p-cymene with key residues.

**Figure 3:**
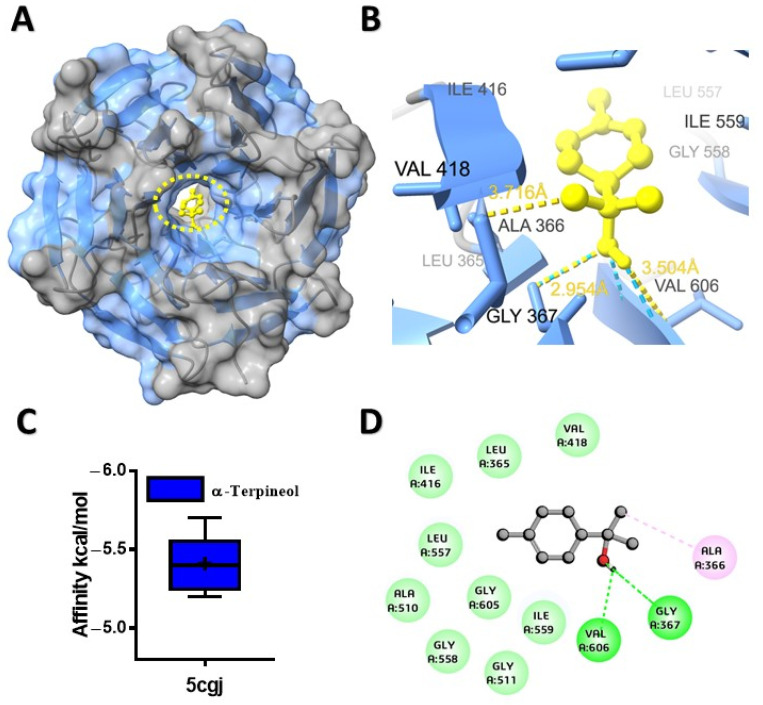
Predicted structures for protein–ligand docked complexes: (**A**) pose view of the interaction of α-terpineol with Nrf2 protein; (**B**) 3D interaction of α-terpineol with Nrf2 protein; (**C**) box plot depicting binding affinity scores for predictions of Nrf2 (5cgj) protein with α-terpineol; (**D**) 2D interaction of α-terpineol with key residues.

**Figure 4:**
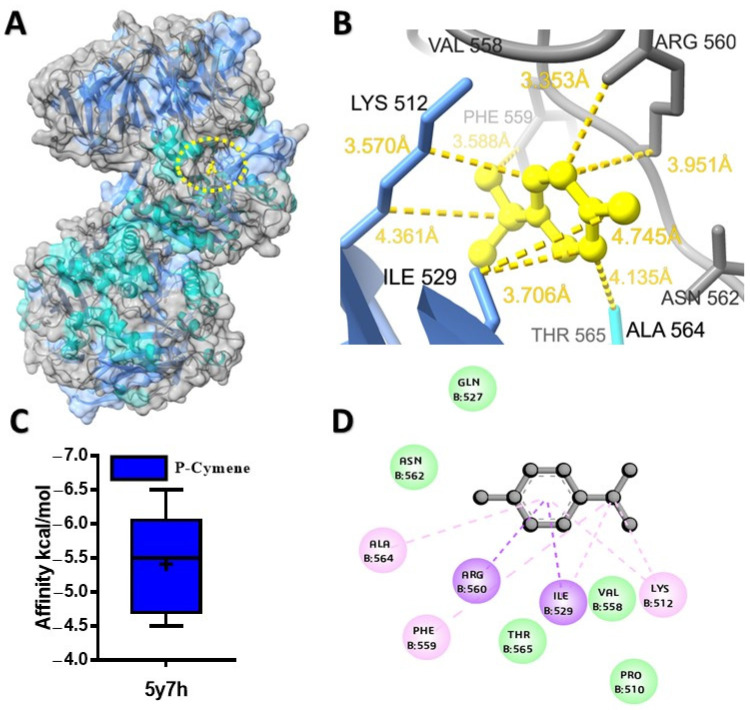
Predicted structures for protein–ligand docked complexes: (**A**) pose view of the interaction of p-cymene with DPP IV protein; (**B**) 3D interaction of p-cymene with DPP IV protein; (**C**) box plot depicting binding affinity scores for predictions of DPP IV (5y7h) protein with p-cymene; (**D**) 2D interaction of p-cymene with key residues.

The potential binding affinity of MQEO towards proteins was shown by the docking score of the complex (Table 1). For all the docking analyses, a higher negative score indicated a better binding affinity. The molecular docking analysis revealed that α-terpineol had the best binding affinity of −6.1 kcal/mol with the cholera toxin protein (Figure 1A). The protein conjugated with α-terpineol resulted in the formation of alkyl/pi-alkyl interactions with chain H (Lys 63), Lys 237, and His 233 of chain C (Figure 1C). We further verified the major constituents of MQEO virtually with 4gfx as an anti-inflammatory target protein. Among the 12 MQEO phytochemical constituents, α-phellandrene and p-cymene showed similar high binding affinities (−5.6 kcal/mol) and interacted in the same pocket with the TXNIP protein (Figure 2A). The best docked pose of α-phellandrene exhibited alkyl and pi-alkyl interactions with 4gfx containing amino acid residues of Tyr 99 and Leu 77 (Figure 2C). The methyl group and aromatic ring of p-cymene recruited Leu 77 by forming alkyl/pi-alkyl interactions; furthermore, the aromatic ring of p-cymene interacted with Tyr 99 (Figure 2F). Among the MQEO constituents, α-terpineol exhibited the highest binding affinity with the Nrf2 protein. α-terpineol showed a conventional hydrogen bond with Val 606 and Gly 367 of chain A, and the rest of the key residues interacted with the alkyl group with Ala 366 (Figure 3B). All the highest binding modes were represented in separate root-mean-square deviation (RMSD), lower-bound (l.b.), and upper-bound (u.b.) values for both the RMSD u.b. and RMSD l.b. groups (Table S1). Of all the proteins currently being studied, MQEO chemical constituents have the highest binding affinities with DPP IV human protein. Crystallized p-cymene revealed interactions with hydrophobic residues of amino acids on the 5y7h protein via associations with the alkyl, pi-alkyl, and pi-sigma groups, which were detected in the coiled and strand regions of the protein B (Figure 4D).

However, the pi-sigma interaction was formed by the aromatic ring of p-cymene. α-terpineol turned out to be the second best (next to p-cymene), having a binding affinity of −6.3 kcal/mol with the DPP IV protein. We further studied the interaction between DPP IV (6bie) and the chemical constituents of MQEO. α-terpineol exhibited the highest binding affinity for the 6bie protein when compared to the other compounds, and it displayed alkyl and pi–alkyl interactions (Figure S2). The molecular docking analysis also revealed that α-terpineol had the best binding affinity of −6.1 kcal/mol with the cholera toxin protein (Figure 4A). The protein conjugated with α-terpineol resulted in the formation of alkyl/pi-alkyl interactions with chain H (Lys 63), Lys 237, and His 233 of chain C (Figure 1C).

### 2.2. Molecular Dynamic Simulation (MD)

**Figure 5:**
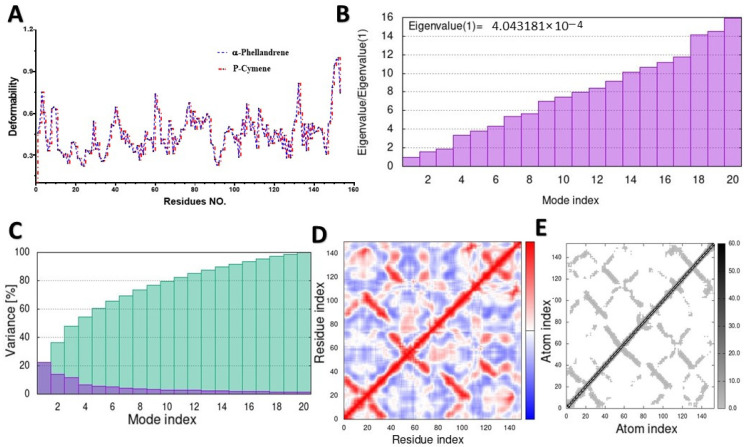
Molecular dynamic simulation of a predicted TXNIP with α-phellandrene: (**A**) deformability; (**B**) eigenvalues; (**C**) variance (blue: individual variances; green: cumulative variances); (**D**) covariance map (residues with correlated motions in red, uncorrelated motions in white, and anticorrelated motions in blue); (**E**) elastic network analysis.

**Figure 6:**
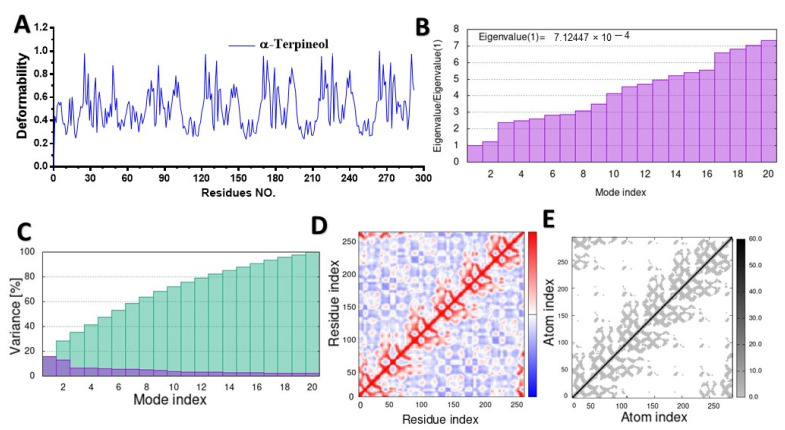
Molecular dynamic simulation of a predicted Nrf2 with α-terpineol: (**A**) deformability; (**B**) eigenvalues; (**C**) variance (blue: individual variances; green: cumulative variances); (**D**) covariance map (residues with correlated motions in red, uncorrelated motions in white, and anticorrelated motions in blue); (**E**) elastic network analysis.

**Figure 7:**
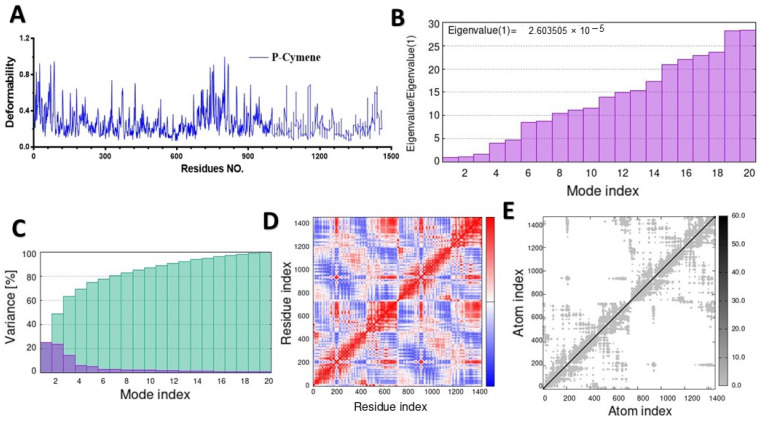
Molecular dynamic simulation of a predicted DPP IV (5y7h) with p-cymene: (**A**) deformability; (**B**) eigenvalues; (**C**) variance (blue: individual variances; green: cumulative variances); (**D**) covariance map (residues with correlated motions in red, uncorrelated motions in white, and anticorrelated motions in blue); (**E**) elastic network analysis.

MD simulation was used to study the behavior of the hinge structures at the atomic level, and it provided a better understanding of how the structure behaves under different loading conditions. The main-chain deformability graph revealed the flexibility of each amino acid residue, with most residues being medium–highly flexible, and they can be easily bent or twisted at the hinge-pointed regions between the ligand and targeted protein (Figure 5A, Figure 6A, Figure 7A, and Figure S3A). The stiffness of a molecule’s motion was represented by the eigenvalues of its normal modes. The docked 4gfx protein with ligand complexes showed the lowest eigenvalues, and it is predicted to be the least deformable and to require minimum energy to change its shape (Figure 5B).

The eigenvalues of the docked complexes 5cjg- α-Terpineol, 5y7h-p-cymene, and 6bie-α-terpineol are shown in Figure 6B, Figure 7B and Figure S3B, respectively. To distinguish between individual and cumulative variances, both are represented by blue and green bars, respectively (Figure 5C). The variance plot of α-terpineol with the target protein showed that the individual variance level was high (22%) (Figure 6C), while the 5cgj protein showed the lowest variance (16%) (Figure 6C).

The covariance map was estimated via the Cartesian coordinates (Cα). The covariance matrices of the docked proteins display stronger associations (red color) with few anticorrelations (blue color) among all the residues (Figure 5D), whereas the white color indicates uncorrelated motion. Likewise, the covariance matrix in Figure 7D represents the moderate correlation between pairs of residues in the protein–ligand complex. The covariance matrices of α-terpineol on 6bie are given in the Supplementary Materials (Figure S3D). In the elastic network model shown in Figure 5E, verified atoms in a protein are connected to each other by springs, which indicates that the stiffness of the target TXNIP protein was very low, as a light-gray color can be observed; therefore, this protein is able to deform easily. Figure 7E and Figure S3E specify a darker-gray portion, as displayed by the pairs of atoms connected by springs in accordance with their stiffer springs.

### 2.3. In Silico Prediction of ADME Properties and Toxicity Parameters of Selected Ligands

For the drug-likeness, we followed Lipinski’s rule of five (RO5). The rules were as follows: molecular weight (MW) < 500; number of H-bond donors < 5; number of hydrogen bond acceptors < 10; Log *p* value < 5; and a molar refractivity range from 40 to 130. Based on these findings, all the lowermost free-binding energy compounds with proteins passed Lipinski’s rules (Table 2). Most of the MQEO compounds did not reveal fatal, toxic, or harmful molecules, and they belong to classes 5 and 6, except for the LD50 of p-cymene (lower than 300 mg/kg), which was predicted to be fatal if swallowed (Table 3). Additionally, all compounds exhibited zero TPSA values. The MQEO compounds have predicted pharmacokinetic profiles and displayed the ability to be easily absorbed through the GI tract and to permeate through the BBB (Table S2). The results of the organ toxicity prediction are shown in Table S3, indicating the MQEO compounds that were mostly nontoxic, except for the carcinogenicity of p-cymene and acetic acid octyl ester, which are considered to be active. The results represented in Table S4 show the highest binding affinities of the MQEO compounds predicted on nuclear receptor signaling pathways and stress response pathways and reveal nontoxic effects.

## 3. Discussion

Molecular docking and MD simulation are two powerful in silico structure-based methods and the most popular and widely used methods for a detailed understanding of the interaction between molecules and proteins at the atomic level. By targeting biological molecules that cause disease, these methods can be used to identify and design new drugs; moreover, these methods are able to facilitate the development of more specific therapeutic agents [42]. The ADME analysis and pharmacokinetic property tools are used to identify any potential risks associated with the physiochemical properties, drug-likeness, lipophilicity, and toxicity of the compound. In this study, all the demonstrated MQEO compounds showed affinities with different proteins; thus, we emphasize that MQEO potentially interacts with all the key proteins involved in DN complications.

In the current investigation, an analysis was conducted on twelve chemical constituents derived from MQEO in terms of their interaction with cholera toxin and the target proteins associated with diabetic nephropathy, namely, TXNIP, Nrf2, and DPP IV. Subsequently, a comprehensive evaluation encompassing molecular dynamic simulations, the drug-like properties, and the ADMET characteristics was performed. Remarkably, α-terpineol displayed notably strong binding affinities towards the cholera toxin protein. In the literature, in a computational study, a screening process was employed to assess the inhibitory potential of 70 herbal compounds against *Vibrio cholerae*. Among the screened compounds, catechin and luteolin emerged as the most promising candidates, exhibiting superior inhibitory properties against the ToxT protein. These two compounds also demonstrated favorable ADME and drug-likeness characteristics, making them prime candidates for future drug development endeavors [43].

The development of gradual kidney dysfunction has been established as a belated and frequently lethal consequence arising from Asiatic cholera and acute renal failure during severe episodes of diarrhea induced by *Vibrio cholerae* [44]. Infections caused by certain strains of *V. cholerae*, such as *V. cholerae* 0139 Bengal, are linked to intestinal inflammation and damage to the tissue [45]. According to our findings, α-terpineol shows high binding affinities toward the cholera toxin protein. A scientific report mentioned the atomistic interaction between cholera toxins and Ganglioside GM1-enriched lipid membranes in the context of Asiatic cholera. This interaction is proposed to offer insights into a plausible mechanism concerning the initial stage of lipid structure and dynamic alterations, which, in turn, facilitate the penetration of cholera toxins into the membrane surface [6]. Furthermore, a comprehensive evaluation of ADMET indicated that cedrelanol has the capability to traverse the gastrointestinal tract and the blood–brain barrier. In the context of antibacterial activity and its potential to inhibit cholera toxin-induced intestinal hypersecretion, it is worth noting that the sesquiterpene T-cadinol, isolated from the aromatic resin of *Commiphora guidottii* (Burseraceae), commonly referred to as scented myrrh, has been documented [46]. Cholera infection is sometimes characterized by the presence of severe electrolyte imbalances and acute kidney injury or inflammation. The occurrence of acute kidney injury may be attributed to renal ischemia, possibly stemming from dehydration, which subsequently results in acute tubular necrosis [47]. In diabetic patients, increased TXNIP expression has been linked to changes in the kidney architecture and function in both diabetes patients and animals [48,49]. Despite the fact that higher levels of serum TXNIP are observed in the renal tissues of diabetic kidney disease patients [50,51], drugs are currently not readily available to control DN development through the suppression of TXNIP. Therefore, our work highlights the potential of TXNIP as a therapeutic target and TXNIP inhibitors as novel therapeutic drugs for targeting DN complications.

Our in silico study revealed, for the first time, the beneficial effect of 12 phytochemical constituents of Maqian that interacted with particular proteins at the molecular level. Through an in vivo study, we previously found that MQEO normalized STZ-induced changes in the kidney protein expressions of TXNIP, IKKβ, and Nrf2 in diabetic mice. We demonstrated that MQEO exerts a potent renoprotective effect by alleviating kidney oxidative stress and inflammation [33]. What is more, MQEO enhanced the liver function by elevating the antioxidant capacity in a type 1 diabetes mellitus mouse model [52].

From the post-docking interaction analysis, the best binding affinities and bosses of the 2D and 3D interactions of ligands with TXNIP were elucidated, which play a vital role in increasing the overall binding energy. Overall, we notice that the common shared binding pocket mostly forms hydrophobic contacts in the absence of hydrogen bonds between these ligands and the TXNIP protein. The hydrophobic nature of phellandrene and p-cymene allows them to easily penetrate cell membranes and subsequently lead to various effects, such as anti-inflammatory activities. The stability and physical movements of the docked complexes were assessed through MD simulations using the iMOD server. The conformationally stable complex of the ligand was observed through a protein-specific pocket site.

The eigenvalues of a normal mode are a measure of how much energy is required to excite that mode. Thus, the higher the eigenvalue, the more energy is required to excite the mode, and the stiffer the molecule in the mode. If the eigenvalue obtained for the 4gfx complex is relatively low and enterprises have low deformability, then this indicates that the interactions are very strong and stable correspondingly. Therefore, we suggest that a stable complex was formed. Furthermore, the covariance map shows that this complex had a comparatively higher number of low and non-interactive residues than high interactive residues, as it indicates correlations between pairs of residues in a protein-docked complex. The elastic map shows how the energy of a protein changes as it is deformed. Most proteins can be affected by changes in their structure. The 4gfx complex displayed pairs of atoms connected by springs in accordance with their lowest degree of stiffness, indicating that this protein can be easily deformed in comparison to the 5y7h and 6bie proteins. Our molecular docking results demonstrated that MQEO containing phellandrene, p-cymene, and other active chemical constituents could be a potent inhibitor of the TXNIP protein. Further mechanistic studies are warranted.

MQEO delays the progression of DN, and the underlying mechanism is probably associated with regulating the Nrf2 pathway [33]. Through the activation of and improvement in the antioxidant capacity of targeted organs, Nrf2 as a factor promotes all anti-inflammatory processes and exhibits a renoprotective effect. In this study, we highlighted the potential roles that the phytochemical constituents from MQEO play in binding with the Nrf2 protein using molecular docking techniques. In terms of cooperation between hydrogen bonds, alkyl hydrophobic bonds, and aromatic ring interactions, it was revealed that docked complexes reinforce each other, and this could exhibit a significant interaction of α-terpineol with protein, showing the compound potentiality to bind affinities at the active site, thereby leading to nuclear translocation and anti-inflammatory activities of Nrf2. The highest binding affinities of α-terpineol were also supported by molecular docking simulation studies on Nrf2 amino acid residues.

The deformable pocket in the protein model was not vigorous, and hence, the protein structure remained quite stable. The associated variance is inversely related to the eigenvalue in normal mode. However, the lower eigenvalues resulting from the Nrf2 protein with the ligand indicated their capability to be deformed. The molecular dynamic simulation results suggest that our complex is plausible and very stable. In this work, and based on our previous in vivo study, we demonstrated that this component binds to the functional amino acid residues of the Nrf2 protein and stimulates its activities; therefore, α-terpineol could be promising as an alternative therapeutic drug for diabetic nephropathy complications through its antioxidant and anti-inflammatory effects by binding with Nrf2.

A docking method was evaluated to predict the efficacy binding strength between 12 MQEO constituents and DPP IV receptor proteins. Our results show that all the compounds had the lowermost free-binding energies docked with the DPP IV protein, as compared to the other proteins under study. DPP IV was shown to interact with remarkable moderate stability and high affinity with the target ligands, as it showed the highest eigenvalue with 23% variance and −6.5 kcal/mol. The observed differences in the binding energies between the two DPP IV proteins raise intriguing questions about their structural and functional characteristics. These structural differences could influence the binding pockets of the two enzymes, leading to different affinities for various compounds. Understanding the functional implications of the binding energy variations could provide valuable insights into the roles of DPP IV enzymes in various biological processes. Inhibitors of the enzyme DPP IV exist in the kidney and are considered as hypoglycemic therapeutic agents. Nevertheless, the impact of the DPP IV protein and the mechanisms underlying its renoprotection on DN patients remain unclear [53]. In diabetes mellitus, in which inflammation is increased, the circulating level of DPP IV is high and the cell membrane associated with the DPP IV is increased [53,54]. Therefore, it is not surprising that MQEO exhibited potent anti-diabetic effects, as we previously reported, as the constituents bind DDP IV strongly and act as DDP IV inhibitors. However, further studies are warranted to identify the exact active ligand and explore more mechanisms of MQEO related to tissue protection in diabetes mellitus.

In this research, chemo-computational, drug-likeness, and toxicology evaluations were performed to predict the cytotoxicity of *Z. myriacanthum* var. *pubescens* Huang essential oil compounds. MQEO small-molecule constituents passed the Lipinski rule of five, thereby indicating that they possess decently high bioavailability with penetration, favorable drug-likeness, and lead-like properties. Moreover, these compounds have been classified into several classes of toxicity according to FDA-approved drugs and using a globally harmonized system (GHS) of chemical labeling classification [55]. The ProTox-II tool was used to divide the compounds into different classes based on their lethal doses upon swallowing. Interestingly, the absence of hepatotoxicity, mutagenicity, and low cytotoxicity was observed in practically all the tested MQEO compounds. Almost all the MQEO compounds showed no interaction with the nuclear receptor signaling pathways or stress response pathways, except n-decanal, which was predicted to interact with estrogen receptor alpha. Further in vivo studies are warranted to assess their potential toxicity effects. Therefore, MQEO constituents show therapeutic potential as promising bioactive compounds for developing new anti-cholera agents and anti-inflammatory renoprotective drugs.

## 4. Materials and Methods

### 4.1. Retrieval and Preparation of Target Receptor Proteins

From the protein data bank repository (https://www.rcsb.org/) (accessed on 16 May 2023), four-dimensional crystal structures of cholera toxin (PDB: 1xtc), TXNIP (PDB: 4gfx), Nrf2 (PDB: 5cgj), and human DPP IV (PDB: 5y7h) and DPP IV (PDB: 6bie) were retrieved, along with co-crystallized ligands. We prepared protein structures for structure-based virtual screening and molecular docking by deleting ligands and water molecules and adding polar hydrogen atoms and other heteroatoms using UCSF Chimera X software version 1.6.1 [56]. An improved PDB file was then utilized to simulate docking.

### 4.2. Ligand Preparation

Our lab previously investigated the chemical compositions of Maqian fruit essential oil, and several components were elucidated in vitro and in vivo [33,57]. A total of 12 major phytochemical compounds were selected for molecular docking. The targeted ligands were retrieved from the PubChem-NCBI database as a structure data file (SDF) (https://pubchem.ncbi.nlm.nih.gov/) (accessed on 17 May 2023). The selected compounds were α-pinene (CID: 6654); β-pinene (CID: 14896); α-phellandrene (CID: 7460); 3-carene (CID: 26049); p-cymene (CID: 7463); d-limonene (CID: 440917); β-phellandrene (CID: 405237423); cis-β-ocimene (CID: 249959750); trans-β-ocimene (CID: 249959748); α-terpineol (CID: 253657446); n-decanal (CID: 8175); and acetic acid octyl ester (CID: 8164). Ligand structures were acquired for optimized chemical structures and minimized energy using Avogadro software version 1.2.0 [58] with the specified Merck Molecular Force Field (MMFF), 1000 steps, the Conjugate Gradient algorithm, and a convergence criterion of 10^−6^. The chemical structures were saved in the MOL2 file.

### 4.3. Molecular Docking

The dockings of small molecules (ligands) with macromolecules (receptors) were uploaded to the free CB-Dock web server (http://clab.labshare.cn/cb-dock/php/index.php) (accessed on 14 June 2023). After submission, both files were converted to pdbqt format using OpenBable (version 3.1.1) and MGLTools version 1.5.7 [59,60], respectively, and submitted, which Autodock Vina worked in the background. Blind docking was performed using CB-DocK, which uses a curvature-based cavity detection approach to identify binding sites and a scoring function to rank the binding poses of ligands [61]. We evaluated the most stable conformation with the highest energy value of each compound for binding the amino acid residue of the protein. For the post-docking analyses and to generate 2D and 3D images, the interactions were visualized using Discovery Studio Visualizer version 21.1.0.20298 and UCSF Chimera X software version 1.6.1, respectively.

### 4.4. Molecular Dynamic Simulation

Based on the binding affinity, we selected the geometries of all protein–ligand complexes for molecular dynamic (MD) simulation. Docked small molecules with macromolecules have been uploaded to the iMODS server (https://bio.tools/imods) (accessed on 30 June 2023). For conforming the stability and to elucidate the predicting properties, such as the main-chain deformability, mobility profiles, eigenvalues, variance, covariance matrix, and elastic network model, normal-mode analysis (NMA) was performed on the protein–ligand interactions [62]. All graphs were generated using GraphPad Prism 5 (GraphPad Software, San Diego, CA, USA).

### 4.5. Prediction of ADME Property and Toxicity Parameters of Selected Ligands

To predict the drug-likeness and pharmacokinetic profiles (absorption, distribution, metabolism, and excretion (ADME)) of the selected compounds according to the high-ranked binding energy scores, we used a free web tool developed by the Swiss Institute of Bioinformatics at www.swissadme.ch [63] (accessed on 7 August 2023). Compound structures were uploaded as SDF files. Physiochemical properties (molecular weight (MW), molecular refractivity (MR), atom count, and topological polar surface area (TPSA)) were computed. Furthermore, the pharmacokinetics was explored for the toxicity properties of the potential MQEO activity prediction using the ProTox-II web server (https://tox-new.charite.de/protox_II/index.php?site=compound_input) (accessed on 8 August 2023) [55]. Before being uploaded to the server, these legends were converted into the simplified molecular input line entry system (SMILES) format.

## 5. Conclusions

In this investigation, a computational approach was employed to assess the potential of various small molecules in Maqian essential oil to target proteins implicated in diabetic nephropathy and inflammation. The results indicate that at least one of the constituents in MQEO holds promise for mitigating inflammation and modifying its impact on diabetic nephropathy complications. Nevertheless, additional research is imperative to delve into the diverse mechanisms underlying MQEO’s potential as a novel renoprotective drug. Our computational analyses brought to light the substantial therapeutic potential of MQEO constituents as candidates for addressing the formidable challenges associated with cholera toxin and diabetic nephropathy. These findings augur well for the future of drug development, personalized medicine, and complementary therapies. Specifically, constituents like α-terpineol, α-phellandrene, and p-cymene within MQEO could lay the groundwork for groundbreaking medications, potentially revolutionizing treatment strategies for cholera and diabetic nephropathy. Incorporating these natural compounds into clinical protocols, dietary supplements, and personalized healthcare regimens has the potential to enhance patient care and alleviate the burden of these debilitating conditions. As we embark on the path of preclinical and clinical trials, foster interdisciplinary collaborations, and secure regulatory approvals, MQEO-derived therapies are poised to assume a pivotal role in the advancement of medical science.

## Figures and Tables

**Table 1:** Binding energies (kcal/mol) of MQEO compounds.

Compound Name	4gfx *	5cgj **	5y7h ***	6bie ****	1xtc *****
α-pinene	−4.3	−5.2	−4.8	−4.9	−5.7
β-pinene	−4.2	−5.1	−5	−5	−5.6
α-phellandrene	−5.6	−4.9	−5.3	−5.5	−5.8
3-carene	−4.4	−5.1	−5.1	−4.2	−5.9
p-cymene	−5.6	−5.1	−6.5	−5.4	−5.8
d-limonene	−5.2	−5.1	−6.2	−5.5	−5.6
β-phellandrene	−5.5	−4.9	−6.2	−5	−5.7
cis-β-ocimene	−4.6	−4.7	−5.6	−4.6	−5
trans-β-ocimene	−4.7	−4.8	−5.4	−4.9	−5.1
α-terpineol	−5	−5.7	−6.3	−5.7	−6.1
n-decanal	−4.3	−4.3	−5.1	−4.6	−4.6
acetic acid octyl ester	−4.5	−4.5	−5.2	−4.2	−5.1

Proteins: * 4gfx: TXNIP; ** 5cgj: Nrf2; *** 5y7h: human DPP IV; **** 6bie: DPP IV; ***** 1xtc: cholera toxin proteins.

**Table 2:** Physicochemical properties, lipophilicities, and drug-likenesses of MQEO compounds from SwissADME web server.

Compound Name	Molecular Weight	Hydrogen Bonds		Rotatable Bonds	LogP * (iLogPo/w)	Molar Refractivity	RO5 Violation **
		Donor	Acceptor				
α-pinene	136	0	0	0	2.69	45.99	1
β-pinene	136	0	0	0	2.47	46.04	1
α-phellandrene	136	0	0	1	2.68	49.89	0
3-carene	136	0	0	0	2.65	50.00	1
p-cymene	134	0	0	1	2.46	47.61	1
d-limonene	136	0	0	1	2.53	49.19	0
β-phellandrene	120	0	0	1	2.65	47.12	0
cis-β-ocimene	120	0	0	3	2.91	48.76	0
trans-β-ocimene	120	0	0	3	2.8	48.76	0
α-terpineol	154	1	1	1	2.59	53.05	0
n-decanal	152	0	1	8	3.05	54.98	0
acetic acid octyl ester	172	0	2	8	2.85	55.93	0

* Log P; octanol–water partition coefficient; ** RO5 violations; Lipinski rule of five.

**Table 3:** Predicted acute oral toxicity of MQEO compounds from ProTox-II web server.

Compound Name	LD50 (mg/kg)	Predicted Toxicity Class *	Average Similarity (%)	Prediction Accuracy (%)
α-pinene	4700	5	100	100
β-pinene	4700	5	100	100
α-phellandrene	5700	6	100	100
3-carene	4800	5	100	100
p-cymene	3	1	100	100
d-limonene	4400	5	100	100
β-phellandrene	5000	5	81.55	70.97
cis-β-ocimene	113	3	69.7	68.07
trans-β-ocimene	113	3	69.7	68.07
α-terpineol	2830	5	100	100
n-decanal	5000	5	100	100
acetic acid octyl ester	3000	5	100	100

* Class 1: fatal if swallowed (LD50 ≤ 5); Class 2: fatal if swallowed (5 < LD50 ≤ 50); Class 3: toxic if swallowed (50 < LD50 ≤ 300); Class 4: harmful if swallowed (300 < LD50 ≤ 2000); Class 5: may be harmful if swallowed (2000 < LD50 ≤ 5000); Class 6: nontoxic (LD50 > 5000).

## Data Availability

Data are contained within the article and the Supplementary Materials.

## Supplementary Materials

The following supporting information can be downloaded at https://www.mdpi.com/article/10.3390/molecules28247954/s1, Figure S1. Molecular dynamic simulation of a predicted TXNIP with p-cymene; Figure S2. Predicted structure of protein–ligand docked complex; Figure S3. Molecular dynamic simulation of a predicted DPP IV (6bie) with α-terpineol; Table S1. RMSD* values for the best-docked modes of protein–ligand complexes; Table S2. Pharmacokinetic properties of MQEO compounds from SwissADME web server; Table S3. Organ toxicity prediction of MQEO compounds from ProTox-II web server; Table S4. Nuclear receptor and stress response signaling pathways for MQEO compound prediction from ProTox-II web server.
