# Supplementary material for: Unveiling the Anti-Cholera and Active Diabetic Renoprotective Compounds of Maqian Essential Oil: A Computational and Molecular Dynamics Study
Source: Molecules. 2023 Dec 5;28(24):7954. doi: 10.3390/molecules28247954 (PMC10746104; doi:10.3390/molecules28247954)
Supplement: Supplementary file 1 [file molecules-28-07954-s001.zip › molecules-2709717-supplementary.pdf]

molecules

MDPI

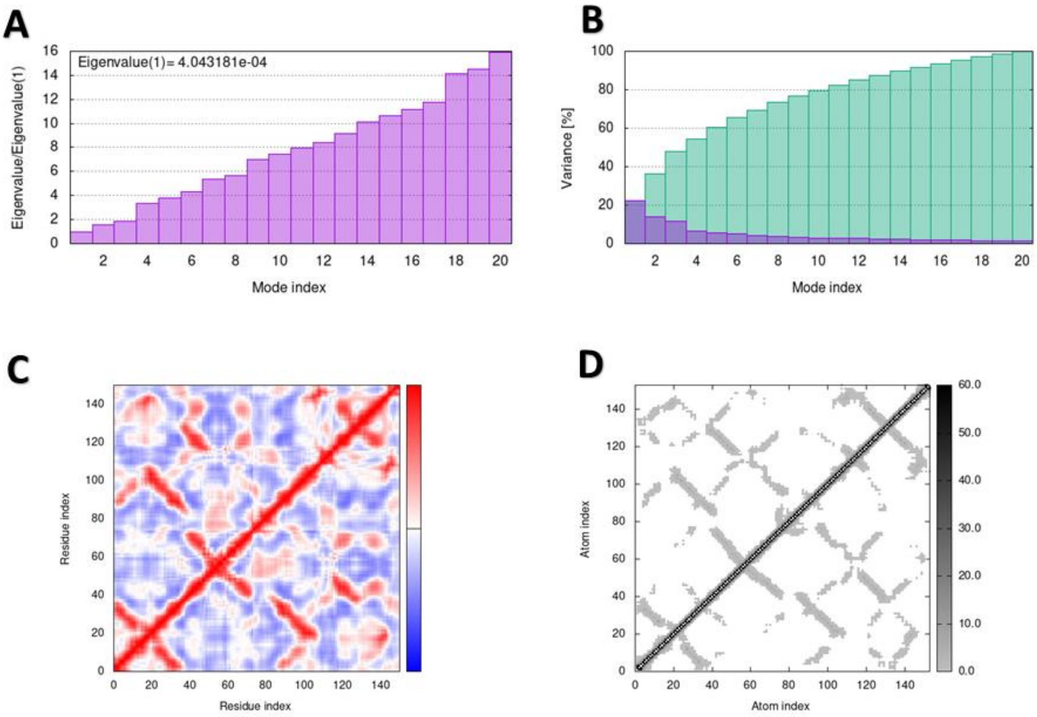

Figure S1. Molecular dynamics simulation of a predicted TXNIP with p-cymene. (A). Deformability, (B) Eigenvalues, (C) Variance (Blue: individual variances, green: cumulative variances), (**D**) Co-variance map (residues with correlated motions in red, uncorrelated motions in white, and anti-correlated motions in blue), (**E**) Elastic network analysis.

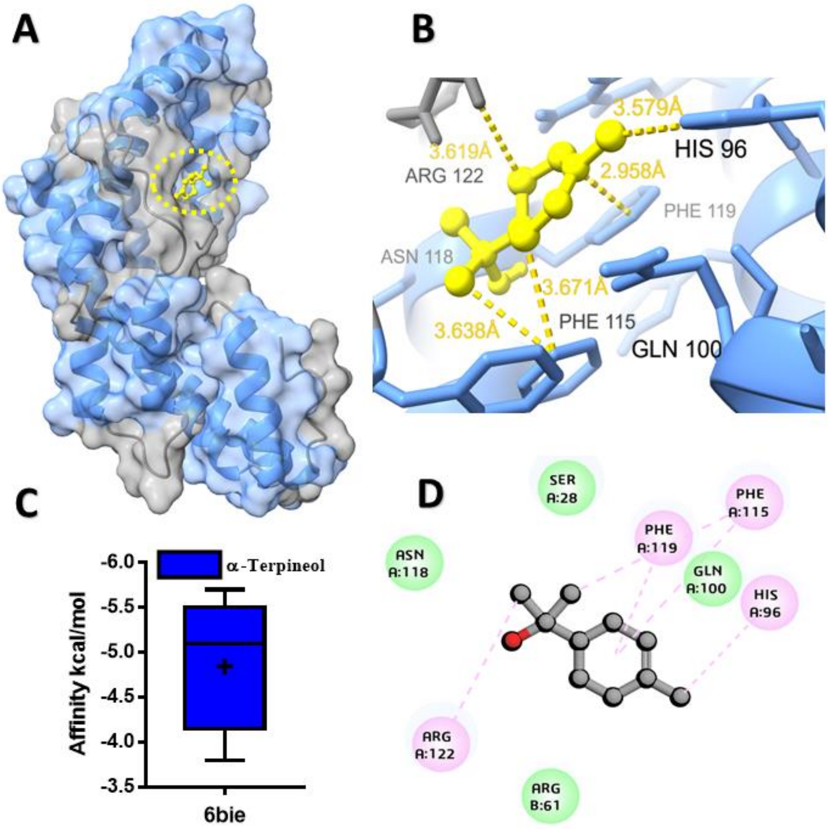

Figure S2. Predicted structure for protein-ligand docked complex. (**A**), Pose view of the interaction of α-Terpineol to the DPP IV protein (**B**) 3D interaction of α-Terpineol with DPP IV protein, (**C**) Box plot depicted binding affinity scores for predictions of DPP IV (6bie) protein with α-Terpineol, (**D**) 2D interaction of α-Terpineol with key residues.

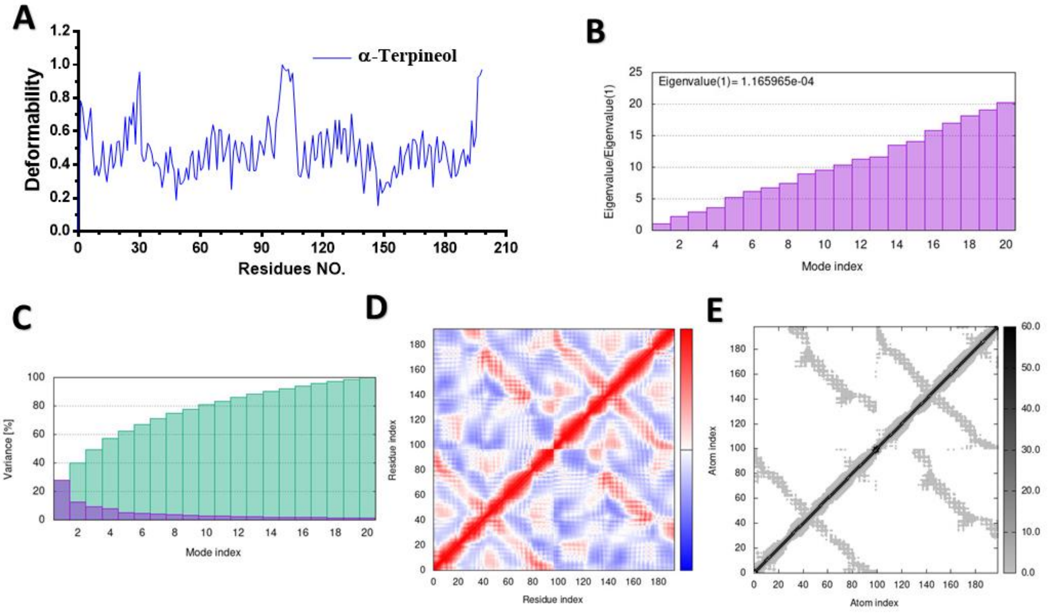

Figure S3. Molecular dynamics simulation of a predicted DPP IV (6bie) with α-Terpineol. (**A**). Deformability, (**B**) Eigenvalues, (**C**) Variance (Blue: individual variances, green: cumulative variances), (**D**) Co-variance map (residues with correlated motions in red, uncorrelated motions in white, and anti-correlated motions in blue), (**E**) Elastic network analysis.

Table S1. RMSD* values for the best docked mode of protein-ligand complexes.

Compound name	4gfx		5cgj		5y7h		6bie		1xtc	
	l.b**	u.b***	l.b	u.b	l.b	u.b	l.b	u.b	l.b	u.b
α-Pinene	0.974	3.407	1.305	3.135	1.139	3.148	1.024	3.420	0.970	3.328
β-Pinene	11.49	13.078	10.851	12.615	1.151	2.054	5.822	7.991	1.069	3.366
α-Phellandrene	**0.883**	**4.315**	10.414	13.099	0.991	4.280	1.231	2.182	1.839	4.306
3-Carene	1.288	3.311	0.807	3.863	1.309	2.795	5.944	8.315	0.814	3.864
p-cymene	1.759	4.342	8.055	8.816	**1.015**	**4.402**	2.313	5.775	1.549	2.174
D-Limonene	2.695	4.558	7.807	8.594	1.317	2.829	1.047	4.358	1.009	4.244
β-Phellandrene	0.911	4.243	13.264	14.803	1.214	4.461	1.318	2.369	1.451	2.239
cis-β-Ocimene	1.610	3.003	3.870	6.648	0.780	5.558	2.044	4.650	1.503	4.371
trans-β-Ocimene	1.758	3.264	2.655	4.664	1.954	3.632	1.894	4.585	7.626	9.728
α-Terpineol	3.602	4.729	**2.471**	**4.671**	1.459	1.792	**2.046**	**4.612**	**1.743**	**2.522**
n-Decana	2.362	4.365	0.978	1.372	2.874	6.288	2.802	5.621	1.807	3.072
Acetic acid octyl ester	3.958	5.514	1.320	3.091	2.691	3.960	2.597	5.569	1.420	2.159

*RMSD; root mean-square deviation for the average distance between the atoms, **l.b; lower bound, ***u.b; upper bound.

Table S2. Pharmacokinetics properties of MQEO compounds from SwissADME web server.

Compound name	GI* absorption	BBB** permeant	Pgp*** substrate	****CYP1A 2 inhibitor	CYP2C19 inhibitor	CYP2C9 inhibitor	CYP2D6 inhibitor	CYP3A4 inhibitor
α-Pinene	Low	Yes	No	No	No	Yes	No	No
β-Pinene	Low	Yes	No	No	No	Yes	No	No
α-Phellandrene	Low	Yes	No	No	No	No	No	No
3-Carene	Low	Yes	No	No	No	Yes	No	No
p-cymene	Low	Yes	No	No	No	No	Yes	No
D-Limonene	Low	Yes	No	No	No	Yes	No	No
β-Phellandrene	Low	Yes	No	No	No	No	No	No
cis-β-Ocimene	Low	Yes	No	No	No	No	No	No
trans-β-Ocimene	Low	Yes	No	No	No	No	No	No
α-Terpineol	High	Yes	No	No	No	No	No	No
n-Decanal	High	Yes	No	No	No	No	No	No
Acetic acid octyl ester	High	Yes	No	No	No	No	No	No

*GI: gastrointestinal, **BBB: blood–brain barrier, ***Pgp: P-glycoprotein, ****CYP: Cytochrome P

Table S3. Organ toxicity prediction of MQEO compound from ProTox-II web server.

Compound name	Hepatotoxicity		Carcinogenicity		Immunotoxicity		Mutagenicity		Cytotoxicity	
	**Pred***	**Prob****	**Pred**	**Prob**	**Pred**	**Prob**	**Pred**	**Prob**	**Pred**	**Prob**
α-Pinene	Inactive	0.8	Inactive	0.66	Inactive	0.97	Inactive	0.95	Inactive	0.71
β-Pinene	Inactive	0.80	Inactive	0.66	Inactive	0.97	Inactive	0.95	Inactive	0.71
α-Phellandrene	Inactive	0.83	Inactive	0.52	Inactive	0.88	Inactive	0.92	Inactive	0.80
3-Carene	Inactive	0.84	Inactive	0.62	Inactive	0.95	Inactive	0.66	Inactive	0.71
p-cymene	Inactive	0.87	**Active**	**0.67**	Inactive	0.99	Inactive	0.98	Inactive	0.89
D-Limonene	Inactive	0.76	Inactive	0.65	Inactive	0.95	Inactive	0.97	Inactive	0.82
β-Phellandrene	Inactive	0.76	Inactive	0.65	Inactive	0.55	Inactive	0.93	Inactive	0.81
cis-β-Ocimene	Inactive	0.83	Inactive	0.51	Inactive	0.99	Inactive	0.90	Inactive	0.75
trans-β-Ocimene	Inactive	0.83	Inactive	0.51	Inactive	0.99	Inactive	0.90	Inactive	0.75
α-Terpineol	Inactive	0.72	Inactive	0.76	Inactive	0.99	Inactive	0.90	Inactive	0.64
n-Decanal	Inactive	0.71	Inactive	0.59	Inactive	0.95	Inactive	0.96	Inactive	0.73
Acetic acid octyl ester	Inactive	0.76	**Active**	**0.56**	Inactive	0.95	Inactive	0.99	Inactive	0.78

*Pred: Prediction, **Prop: Probability.

Table S4. Nuclear receptor and stress response signaling pathways for MQEO compounds prediction from ProTox-II web server.

Compound name	AhR*		AR**		AR-LBD***		Aromatase		ER****		ER-LBD*****	
	Pred	Prob	Pred	Prob	Pred	Prob	Pred	Prob	Pred	Prob	Pred	Prob
α-Pinene	Inactive	0.99	Inactive	0.99	Inactive	0.99	Inactive	0.94	Inactive	0.97	Inactive	0.99
β-Pinene	Inactive	0.99	Inactive	0.99	Inactive	0.99	Inactive	0.94	Inactive	0.97	Inactive	0.99
α-Phellandrene	Inactive	1.0	Inactive	1.0	Inactive	0.99	Inactive	1.0	Inactive	0.97	Inactive	0.99
3-Carene	Inactive	0.99	Inactive	1.0	Inactive	1.0	Inactive	0.95	Inactive	0.95	Inactive	0.99
p-cymene	Inactive	1.00	Inactive	1.00	Inactive	1.00	Inactive	1.00	Inactive	1.00	Inactive	1.00
D-Limonene	Inactive	1.00	Inactive	0.99	Inactive	1.00	Inactive	0.99	Inactive	0.84	Inactive	1.00
β-Phellandrene	Inactive	0.99	Inactive	1.00	Inactive	0.99	Inactive	1.00	Inactive	0.94	Inactive	0.99
cis-β-Ocimene	Inactive	0.99	Inactive	1.00	Inactive	0.99	Inactive	1.00	Inactive	0.97	Inactive	0.99
trans-β-Ocimene	Inactive	0.99	Inactive	1.00	Inactive	0.99	Inactive	1.00	Inactive	0.97	Inactive	0.99
α-Terpineol	Inactive	0.99	Inactive	0.99	Inactive	1.00	Inactive	0.97	Inactive	0.93	Inactive	0.98
n-Decanal	Inactive	1.00	Inactive	1.00	Inactive	1.00	Inactive	1.00	**Active**	0.87	Inactive	1.00
Acetic acid octyl ester	Inactive	1.00	Inactive	1.00	Inactive	1.00	Inactive	1.00	Inactive	0.58	Inactive	1.00

*AhR; Aryl hydrocarbon Receptor, **AR; Androgen Receptor, ***AR-LBD; Androgen Receptor Ligand Binding Domain, ****ER; Estrogen Receptor Alpha, *****ER-LBD; Estrogen Receptor Ligand Binding Domain.

Table S4. Cont. .

Compound name	PPAR-Gamma*		nrf2/ARE**		HSE***		MMP****		p53*****		ATAD5******	
	Pred	Prob	Pred	Prob	Pred	Prob	Pred	Prob	Pred	Prob	Pred	Prob
α-Pinene	Inactive	0.99	Inactive	0.96	Inactive	0.96	Inactive	0.97	Inactive	1.0	Inactive	1.0
β-Pinene	Inactive	0.99	Inactive	0.96	Inactive	0.96	Inactive	0.97	Inactive	1.0	Inactive	1.0
α-Phellandrene	Inactive	1.0	Inactive	0.99	Inactive	0.99	Inactive	0.97	Inactive	1.0	Inactive	1.0
3-Carene	Inactive	0.99	Inactive	0.95	Inactive	0.95	Inactive	0.94	Inactive	0.99	Inactive	0.99
p-cymene	Inactive	1.00	Inactive	1.00	Inactive	1.00	Inactive	1.00	Inactive	1.00	Inactive	1.00
D-Limonene	Inactive	1.00	Inactive	0.98	Inactive	0.98	Inactive	1.00	Inactive	1.00	Inactive	1.00
β-Phellandrene	Inactive	1.00	Inactive	0.70	Inactive	0.70	Inactive	0.96	Inactive	1.00	Inactive	1.00
cis-β-Ocimene	Inactive	1.00	Inactive	0.95	Inactive	0.95	Inactive	0.99	Inactive	1.00	Inactive	1.00
trans-β-Ocimene	Inactive	1.00	Inactive	0.95	Inactive	0.95	Inactive	0.99	Inactive	1.00	Inactive	1.00
α-Terpineol	Inactive	0.99	Inactive	0.97	Inactive	0.97	Inactive	0.92	Inactive	1.00	Inactive	1.00
n-Decanal	Inactive	0.99	Inactive	1.00	Inactive	1.00	Inactive	1.00	Inactive	1.00	Inactive	1.00
Acetic acid octyl ester	Inactive	1.00	Inactive	1.00	Inactive	1.00	Inactive	1.00	Inactive	1.00	Inactive	1.00

*PPAR-Gamma; eroxisome Proliferator Activated Receptor Gamma, **nrf2/ARE: Nuclear factor (erythroid-derived 2)-like 2/antioxidant responsive element, ***HSE; Heat shock factor response element, ****MMP; Mitochondrial Membrane Potential, *****p53; Phosphoprotein (Tumor Supressor), ******ATAD5; ATPase family AAA domain-containing protein 5.
